# Patterns and trends of antibacterial treatment in patients with urinary tract infections, 2015–2019: an analysis of health insurance data

**DOI:** 10.1186/s12875-022-01816-6

**Published:** 2022-08-11

**Authors:** Guido Schmiemann, Falk Hoffmann, Axel Hamprecht, Kathrin Jobski

**Affiliations:** 1 Institute for Public Health and Nursing Sciences, Department for Health Services Research, Bremen University, Grazer Str. 4, 28359 Bremen, Deutschland; 2 Department of Health Services Research, Carl Von Ossietzky University of Oldenburg, Ammerländer Heerstraße 114-118, 26129 Oldenburg, Germany; 3 Department of Medical Microbiology and Virology, Carl Von Ossietzky University of Oldenburg, Ammerländer Heerstraße 114-118, 26129 Oldenburg, Germany

**Keywords:** Guideline adherence, Prescription pattern, Health services research, Primary care, Urology

## Abstract

**Background:**

Urinary tract infections are among the most common reason for encounter and subsequent antibiotic prescriptions. Due to the risk of collateral damage and increasing resistance rates, explicit recommendations against the use of fluoroquinolones like ciprofloxacin in uncomplicated urinary tract infections have been issued. However, to what extent these recommendations were followed and if there are relevant differences between the disciplines involved (general practitioners, urologists, paediatricians and gynaecologists) are unknown.

**Methods:**

We used anonymized data from a local statutory health insurance (SHI) company, which covered about 38% of all SHI-insured persons in the federal state of Bremen, Germany between 2015—2019. Data included demographics, outpatient diagnoses and filled prescriptions on an individual level.

**Results:**

One-year prevalence of urinary tract infections was 5.8% in 2015 (females: 9.2%, males: 2.5%). Of all 102,715 UTI cases, 78.6% referred to females and 21.4% to males, 6.0% of cases were younger than 18 years. In females, general practitioners were the most common diagnosing speciality (52.2%), followed by urologists (20.0%) and gynaecologists (16.1%). Overall, fluoroquinolones were most often prescribed (26.3%), followed by fosfomycin (16.1%) and the combination of sulfamethoxazole and trimethoprim (14.2%). Fluoroquinolones were most often prescribed by urologists and general practitioners, while gynaecologists preferred fosfomycin. During the study period, shares of fluoroquinolones decreased from 29.4% to 8.7% in females and from 45.9% to 22.3% in males.

**Conclusions:**

Despite a clear trend toward a more guideline adherent prescription pattern, there is still room for improvement regarding the use of second-line antibiotics especially fluoroquinolones. The choice of antibiotics prescribed differs between specialities with higher uptake of guideline-recommended antibiotics by gynaecologists, mainly because of higher prescription shares of fosfomycin.

**Supplementary Information:**

The online version contains supplementary material available at 10.1186/s12875-022-01816-6.

## Background

Urinary tract infections (UTI) are among the most common reasons for consultations in primary care with a prevalence of nearly 9% per year in women [1, 2]. UTI and upper respiratory tract infections are the most common reasons for antibiotic prescriptions. The majority of patients with UTIs receive an antibiotic as recommended by most European guidelines [3]. However, the inappropriate use of broad-spectrum antibiotics is an important driver for antibiotic resistance, which is a serious public and individual health concern. Additionally, inappropriate antibiotic prescription, in general, is an important contributor to this development.

The implementation of evidence-based guidelines with specific recommendations on antibiotic prescriptions ideally based on local resistance rates can be an important tool to tackle antibiotic resistance and increase the quality of care [4].

In 2000 the first guideline on UTI was published by the German College of General Practitioners and Family Physicians. As patients in Germany have direct access to specialist care, those with UTI symptoms might choose between seeing a General Practitioner (GP) or a gynaecologist/ urologist/paediatrician depending on age, sex and availability. Fortunately, in 2010, the national guideline on UTI was published. This guideline has been developed as an interdisciplinary project with representatives of GPs, urologists and gynaecologists.

This guideline has been updated regularly [5, 6] with few changes in the antibiotics recommended. Since 2010 nitrofurantoin, fosfomycin, pivmecillinam and trimethoprim are recommended for uncomplicated urinary tract infections in adult women. With the 2017 update [6] nitroxoline was additionally recommended as another first-line therapy. In men, pivmecillinam and nitrofurantoin were recommended as a first choice.

Due to the high risk of collateral damage, increasing resistance rates and potential of individual harm (tendinopathy and aortic dissection among others), both international and German guidelines explicitly recommend against the use of fluoroquinolones (FQ) like ciprofloxacin in uncomplicated urinary tract infections [5, 7]. These recommendations were further augmented by safety alerts. The European Medicine Agency (EMA) recommended a restricted use in 2018 and 2019 and as a consequence Dear doctor letters were sent out in Germany to inform physicians individually [8].

However, actual prescription patterns do not necessarily follow guideline recommendations. As the awareness regarding the increasing antimicrobial resistance has grown and the safety concerns regarding inappropriate FQ use have risen [9] current data on prescription patterns are warranted to adapt antibiotic stewardship strategies. According to an analysis of claims data from 2009, ciprofloxacin was still among the top three antibiotics for all indications prescribed by GPs, urologists and gynaecologists in Germany [10]. In 2013 ciprofloxacin was prescribed in 33% of all urinary tract infections [1] but the current prescription habits for UTIs are not known. Therefore, we wanted to analyse current prescription patterns and trends in urinary tract infections and assess if prescriptions differed between GPs, urologists and gynaecologists.

## Methods

### Data source and study population

We used anonymized data from the AOK Bremen/Bremerhaven, a local statutory health insurance (SHI) company, which in 2019 covered about 38% of all SHI-insured persons in the federal state of Bremen [11]. Bremen, the smallest of the 16 German federal states, has about 680,000 inhabitants corresponding to 0.8% of the German population. The study period encompassed the years 2015 to 2019. Data included demographics (sex and birth year) as well as outpatient diagnoses and filled prescriptions on an individual level.

The study population included all persons with valid information on age and sex with at least one diagnosis of UTI as an outpatient during the study period.

### Case definition

UTI diagnoses were defined according to the German modification of the International Classification of Diseases (10th revision) [12] and included: urinary tract infection, site not specified (N39.0), acute cystitis (N30.0), cystitis, unspecified (N30.9) and acute tubulo-interstitial nephritis (N10). This diagnosis by ICD does not allow the differentiation between complicated and uncomplicated infections, often used by guidelines to recommend a specific therapeutic approach.

In Germany, outpatient diagnoses are only reimbursed quarterly (i.e. four three-month periods per year). The data did not allow to determine whether diagnoses from different physicians or diagnoses recorded in the end of one and the beginning of the next quarter referred to one UTI (including follow-up visits) or multiple (separate) infections. Therefore, a UTI case was defined as a person with at least one UTI diagnosis in a respective quarter. Consequently, persons with multiple UTI diagnoses in one quarter were counted as one case only. Infections spanning the turn of a quarter and resulting in physician contacts in both quarters were calculated as two cases. A person could therefore become a UTI case in each quarter of the study period.

For each case, we assessed the respective UTI diagnosis. Cases with different UTI diagnoses in one quarter, irrespective of whether these were recorded by one or more physicians, were classified as having received “multiple diagnoses”. Information on the diagnosing physician included his or her speciality (i.e. about 70 distinct specialities). These specialities were grouped as GP, urologist, gynaecologist, paediatrician, or other. If more than one grouped speciality recorded UTI diagnoses in a quarter, the case was considered diagnosed by “multiple specialities”.

Subgroup analysis included only incident UTI cases defined as those who had no UTI diagnoses in the four quarters preceding a respective quarter while being insured in each of these four quarters. In this analysis, persons could also be included as a case more than one time when further episodes also fulfilled this definition.

### Antibacterial treatment

Antibacterial treatment was based on the anatomical therapeutic chemical (ATC) classification [13]. Prescriptions were classified according to the current German guideline [14] and included beta-lactam antibacterials, penicillins (J01C excl. J01CA08), cephalosporins (J01DB, J01DC, J01DD, J01DE), fluoroquinolones (J01MA), fosfomycin (J01XX01), nitrofurantoin (J01XE01, J01XE51), nitroxoline (J01XX07), pivmecillinam (J01CA08), the combination of sulfamethoxazole and trimethoprim (J01EE01), trimethoprim (J01EA01) and other antibacterials (all other ATC codes starting with J01).

In the data, prescriptions could not be directly linked to a respective diagnosis or the diagnosing physician. Therefore, all prescriptions filled in the quarter of a UTI diagnosis were included. However, two different definitions of antibacterial treatment were applied: First, a UTI case was classified as treated if he or she received at least one antibacterial drug in the quarter of a UTI diagnosis at all. In a second and more narrow definition, a case was only considered treated if an antibacterial drug had been prescribed by at least one speciality also recording a diagnosis in the respective quarter.

### Analysis

First, prevalences of UTI were assessed by year (2015, 2016, 2017, 2018 and 2019), stratified by sex and age group (0–5, 6–13, 14–17, 18–24, followed by seven 10-years age intervals and the interval 95 +). The prevalence was defined as the number of persons with at least one UTI diagnosis in a year divided by all persons of the respective stratum insured for at last one day of that year.

Second, we assessed the number of cases per person (e.g. in how many of the 20 quarters at least a UTI diagnosis was recorded). We also calculated how many persons had recurring UTI diagnoses defined as (1) diagnoses in two consecutive quarters and (2) diagnoses in three of four consecutive quarters.

Third, case characteristics including the UTI diagnosis, the grouped speciality of the diagnosing physician and whether cases received antibacterial treatment were presented by sex and age group (0–5, 6–13, 14–17, 18 + years) using descriptive statistics (frequencies and percentages).

Fourth, time trends of antibacterial prescriptions every quarter (i.e. 20 quarters) were displayed by sex and by the grouped speciality of the prescribing physician.

Fifth, these analyses were rerun for the subgroup of incident UTI cases.

Analyses were performed using SAS, version 9.4 (SAS Institute Inc., Cary, NC).

## Results

### UTI prevalence and recurring UTIs

In total, 102,715 UTI cases were recorded between 2015 and 2019. The UTI prevalence was 5.8% in 2015 (females: 9.2%, males: 2.5%) and 5.1% in 2019 (females: 8.2%, males: 2.1%), Table 1; Supplemental Table 1. With respect to age, in 2019, the prevalence ranged from 1.4% in the youngest group to 12.1% in those aged 85 to 94 years.

**Table 1:** Characteristics of UTI cases from 2015 to 2019 by sex and age group based on all UTI cases during the study period

	**Total**	**Sex**		**Age group**			
	**(*N* = 102,715)**	**Female (*N* = 80,767)**	**Male (*N* = 21,948)**	**0–5 years (*N* = 1,864)**	**6–13 years (*N* = 2,190)**	**14–17 years (*N* = 2,109)**	** > = 18 years (*N* = 96,552)**
**Age group in years**	**(*N* = 102,715)**	**(*N* = 80,767)**	**(*N* = 21,948)**	**(*N* = 1,864)**	**(*N* = 2,190)**	**(*N* = 2,109)**	**(*N* = 96,552)**
0–5	1,864 (1.8%)	1,453 (1.8%)	411 (1.9%)	1,864 (100%)			
6–13	2,190 (2.1%)	1,914 (2.4%)	276 (1.3%)		2,190 (100%)		
14–17	2,109 (2.1%)	1,938 (2.4%)	171 (0.8%)			2,109 (100%)	
18–24	9,131 (8.9%)	8,187 (10.1%)	944 (4.3%)				9,131 (9.5%)
25–34	13,217 (12.9%)	11,462 (14.2%)	1,755 (8.0%)				13,217 (13.7%)
35–44	10,530 (10.3%)	8,794 (10.9%)	1,736 (7.9%)				10,530 (10.9%)
45–54	10,808 (10.5%)	8,424 (10.4%)	2,384 (10.9%)				10,808 (11.2%)
55–64	11,466 (11.2%)	8,540 (10.6%)	2,926 (13.3%)				11,466 (11.9%)
65–74	13,302 (13.0%)	9,606 (11.9%)	3,696 (16.8%)				13,302 (13.8%)
75–84	19,302 (18.8%)	13,858 (17.2%)	5,444 (24.8%)				19,302 (20.0%)
85–94	8,051 (7.8%)	5,971 (7.4%)	2,080 (9.5%)				8,051 (8.3%)
95 +	745 (0.7%)	620 (0.8%)	125 (0.6%)				745 (0.8%)
**Diagnoses**	**(*N* = 102,715)**	**(*N* = 80,767)**	**(*N* = 21,948)**	**(*N* = 1,864)**	**(*N* = 2,190)**	**(*N* = 2,109)**	**(*N* = 96,552)**
N39.0	66,022 (64.3%)	50,881 (63.0%)	15,141 (69.0%)	1,594 (85.5%)	1,787 (81.6%)	1,431 (67.9%)	61,210 (63.4%)
N30.0	14,963 (14.6%)	11,262 (13.9%)	3,701 (16.9%)	59 (3.2%)	116 (5.3%)	218 (10.3%)	14,570 (15.1%)
N30.9	12,961 (12.6%)	11,327 (14.0%)	1,634 (7.4%)	102 (5.5%)	175 (8.0%)	281 (13.3%)	12,403 (12.8%)
N10	950 (0.9%)	776 (1.0%)	174 (0.8%)	53 (2.8%)	23 (1.1%)	13 (0.6%)	861 (0.9%)
Multiple diagnoses	7,819 (7.6%)	6,521 (8.1%)	1,298 (5.9%)	56 (3.0%)	89 (4.1%)	166 (7.9%)	7,508 (7.8%)
**Any antibacterial prescribed?**^a^	**(*N* = 102,715)**	**(*N* = 80,767)**	**(*N* = 21,948)**	**(*N* = 1,864)**	**(*N* = 2,190)**	**(*N* = 2,109)**	**(*N* = 96,552)**
Yes	69,591 (67.8%)	57,206 (70.8%)	12,385 (56.4%)	1,381 (74.1%)	1,625 (74.2%)	1,623 (77.0%)	64,962 (67.3%)
**Grouped diagnosing physician specialty**	**(*N* = 93,412)**	**(*N* = 73,082)**	**(*N* = 20,330)**	**(*N* = 1,678)**	**(*N* = 2,019)**	**(*N* = 1,943)**	**(*N* = 87,772)**
GP	45,975 (49.2%)	38,181 (52.2%)	7,794 (38.3%)	138 (8.2%)	385 (19.1%)	910 (46.8%)	44,542 (50.7%)
Urologist	25,575 (27.4%)	14,631 (20.0%)	10,944 (53.8%)	66 (3.9%)	145 (7.2%)	145 (7.5%)	25,219 (28.7%)
Gynaecologist	11,826 (12.7%)	11,798 (16.1%)	28 (0.1%)	2 (0.1%)	29 (1.4%)	256 (13.2%)	11,539 (13.1%)
Pediatrician	3,519 (3.8%)	3,002 (4.1%)	517 (2.5%)	1,434 (85.5%)	1,402 (69.4%)	508 (26.1%)	175 (0.2%)
Other	1,438 (1.5%)	1,169 (1.6%)	269 (1.3%)	12 (0.7%)	7 (0.3%)	25 (1.3%)	1,394 (1.6%)
Multiple specialties	5,079 (5.4%)	4,301 (5.9%)	778 (3.8%)	26 (1.5%)	51 (2.5%)	99 (5.1%)	4,903 (5.6%)
**Any antibacterial prescribed by diagnosing specialty?**^b^	**(*N* = 93,412)**	**(*N* = 73,082)**	**(*N* = 20,330)**	**(*N* = 1,678)**	**(*N* = 2,019)**	**(*N* = 1,943)**	**(*N* = 87,772)**
Yes	57,993 (62.1%)	48,165 (65.9%)	9,828 (48.3%)	1,210 (72.1%)	1,469 (72.8%)	1,438 (74.0%)	53,876 (61.4%)

^a^in the quarter of a UTI diagnosis, by any physician

^b^in the quarter of a UTI diagnosis, by a physician specialty diagnosing a UTI in the respective quarter

*UTI* Urinary tract infection

The 102,715 UTI cases referred to 47,396 persons with at least one UTI diagnosis during the study period. Of those persons, 41.3% received UTI diagnoses in more than one quarter (females more often than males), 22.0% had diagnoses in two consecutive quarters and 11.4% were diagnosed in at least three of four consecutive quarters (Supplemental Table 2).

### Characteristics of UTI cases

Of all 102,715 UTI cases, 78.6% referred to females and 21.4% to males; 6.0% of cases were observed in patients younger than 18 years. The most common diagnosis was a “urinary tract infection, site not specified” (64.3% overall, > 80% in those up to the age of 13). In females, GPs were the most common diagnosing specialty (52.2%), followed by urologists (20.0%) and gynaecologists (16.1%), whereas male patients most often received their diagnosis from urologists (53.8%) and GPs (38.3%). Up to the age of 13 years, paediatricians were the most common diagnosing speciality.

Antibacterials were prescribed in 70.8% of female and 56.4% of male cases. In patients up to 17 years, higher proportions of antibacterial treatment were recorded (> 74%) than in those aged 18 years and older (67.3%). When considering only prescriptions that have been issued by the physician specialties (GP, paediatrician, urologist, gynaecologist) who had documented a UTI diagnosis in the respective quarter, 65.9% of females and 48.3% of males received antibacterial treatment. This proportion further increased if only incident cases were considered (see below).

### Antibacterial treatment by age and sex

In total, 102,006 antibacterial prescriptions were issued in quarters with UTI diagnoses. Overall, fluoroquinolones (FQ) were most often prescribed (26.3%), followed by fosfomycin (16.1%) and the combination of sulfamethoxazole and trimethoprim (co-trimoxazole, 14.2%).

**Fig. 1:**
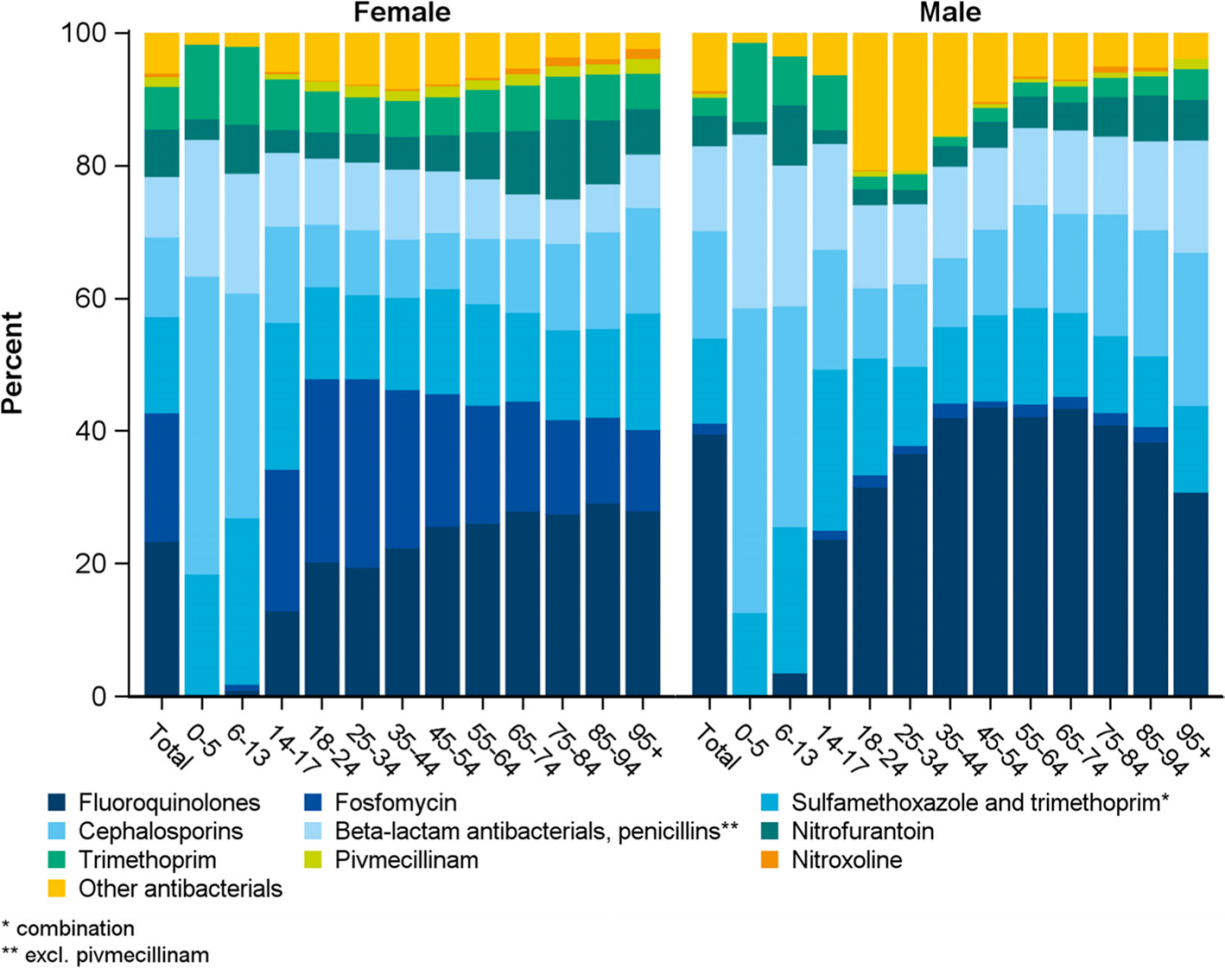
Shares of antibacterials prescribed in the quarter of a UTI diagnosis from 2015 to 2019 by sex and age group. (*N* = 102,006 prescriptions)

In females, most commonly prescribed antibiotics were FQ (23.3%) and fosfomycin (19.4%), whereas in male cases FQ dominated (39.5%), followed by (far less) prescriptions of cephalosporins (16.2%, Fig. 1).

Up to the age of 13, cephalosporins, penicillins and co-trimoxazole accounted for more than three quarters of prescriptions for both sexes. In females aged 14 to 44, fosfomycin was the most commonly prescribed antibacterial, whereas FQ dominated prescriptions in those aged 45 and older. In male patients, FQ were the most common antibacterials in all groups aged 18 years or older. Use increased with rising age but decreased in cases aged 75 or older.

### Trends in antibacterial treatment

**Fig. 2:**
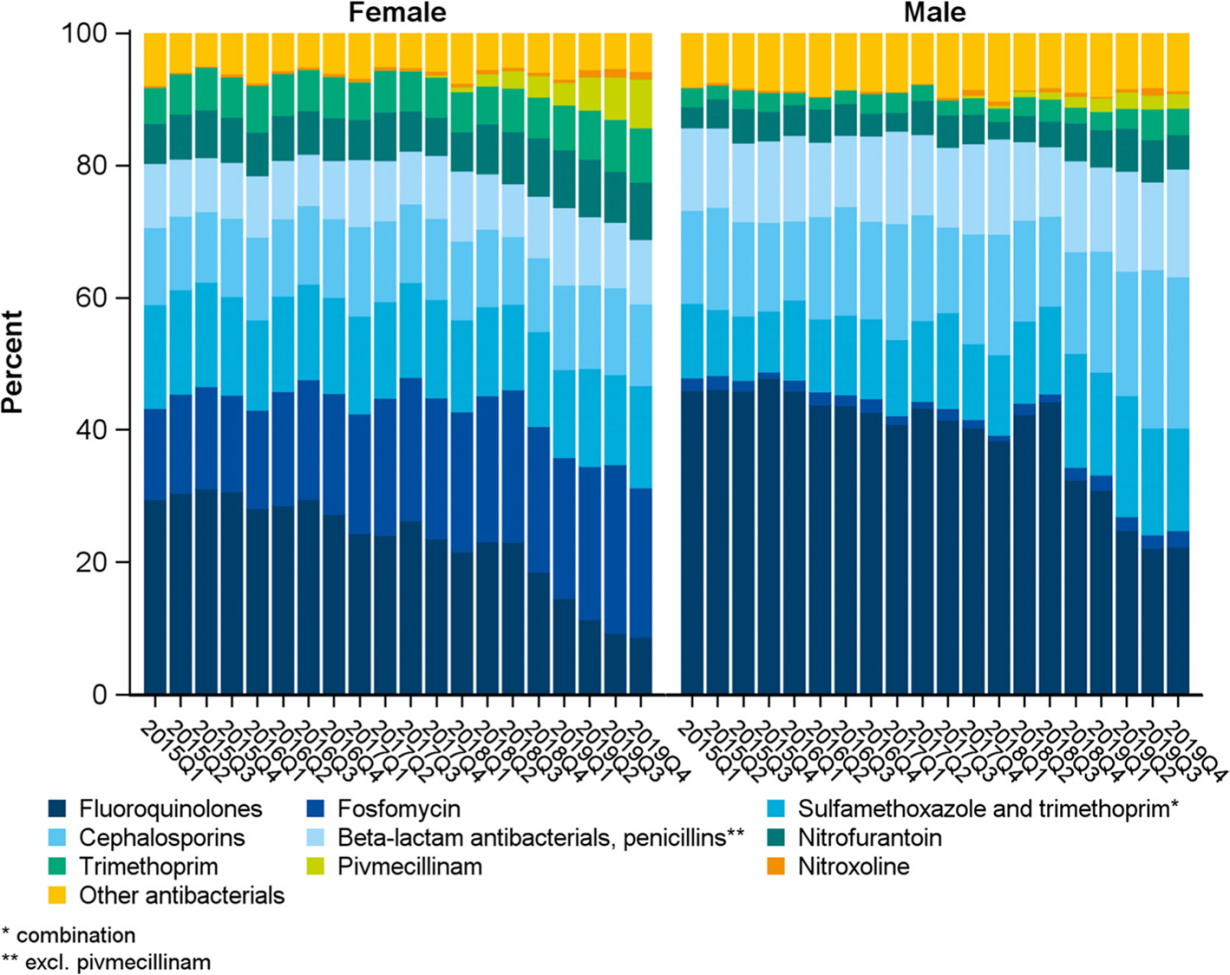
Trend: shares of antibacterials prescribed in the quarter of a UTI diagnosis by sex. (*N* = 102,006 prescriptions)

During the study period, shares of FQ decreased in females (from 29.4% in the first quarter of 2015 to 8.7% in the last quarter of 2019) and in males from 45.9% to 22.3%, Fig. 2). In females, the largest increase was observed for fosfomycin (13.8% to 22.6%) and pivmecillinam (reaching 7.4% at the end of the study period). In males, cephalosporins (14.1% to 22.9%) and, to a lesser extent, penicillins and co-trimoxazole compensated the decrease of FQ.

### Antibacterial treatment and trends by physician speciality

**Fig. 3:**
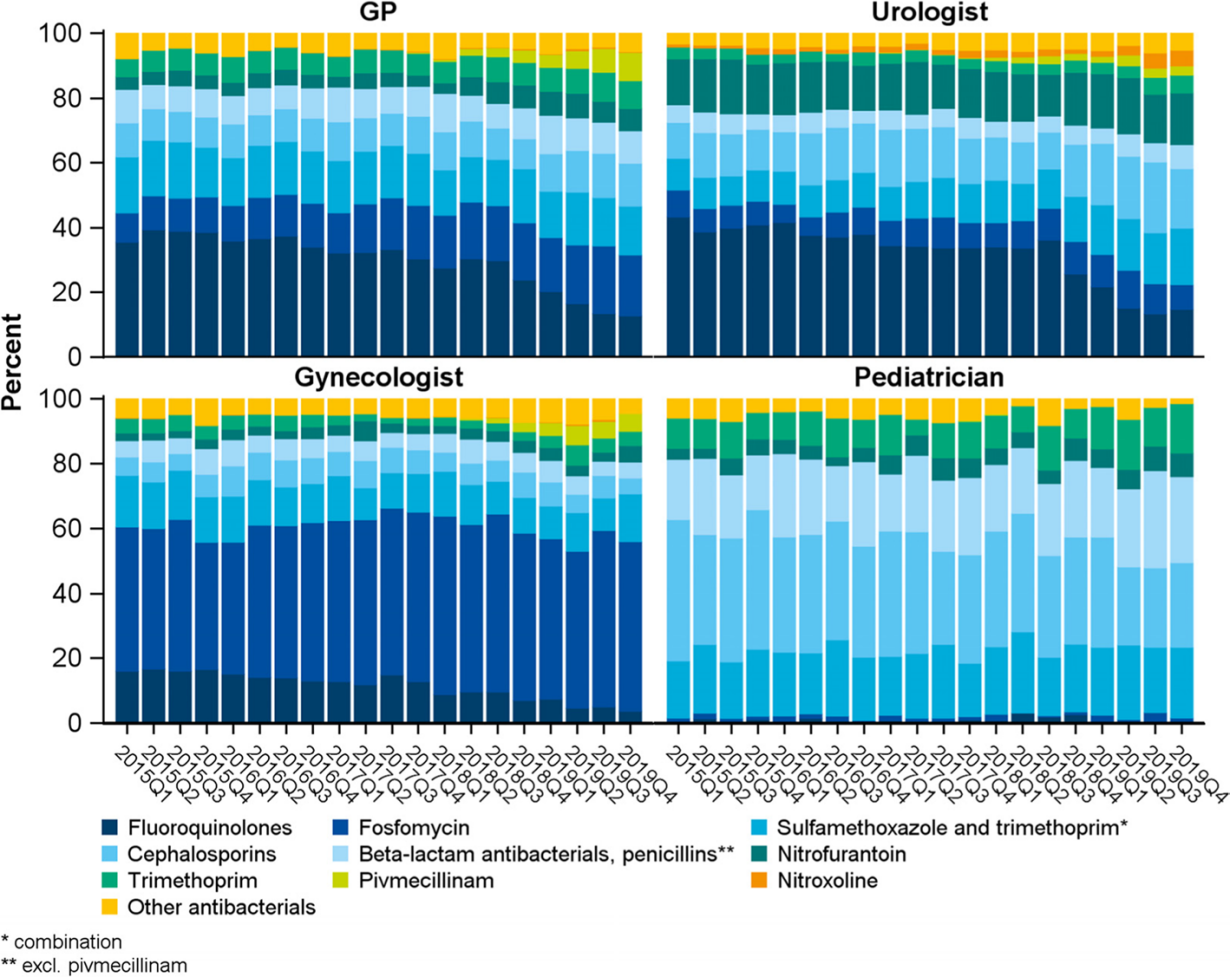
Trend: shares of antibacterials prescribed by GP, urologist, gynaecologist and paediatrician in the quarter of a UTI diagnosis. (*N* = 95,648 prescriptions)

**Fig. 4:**
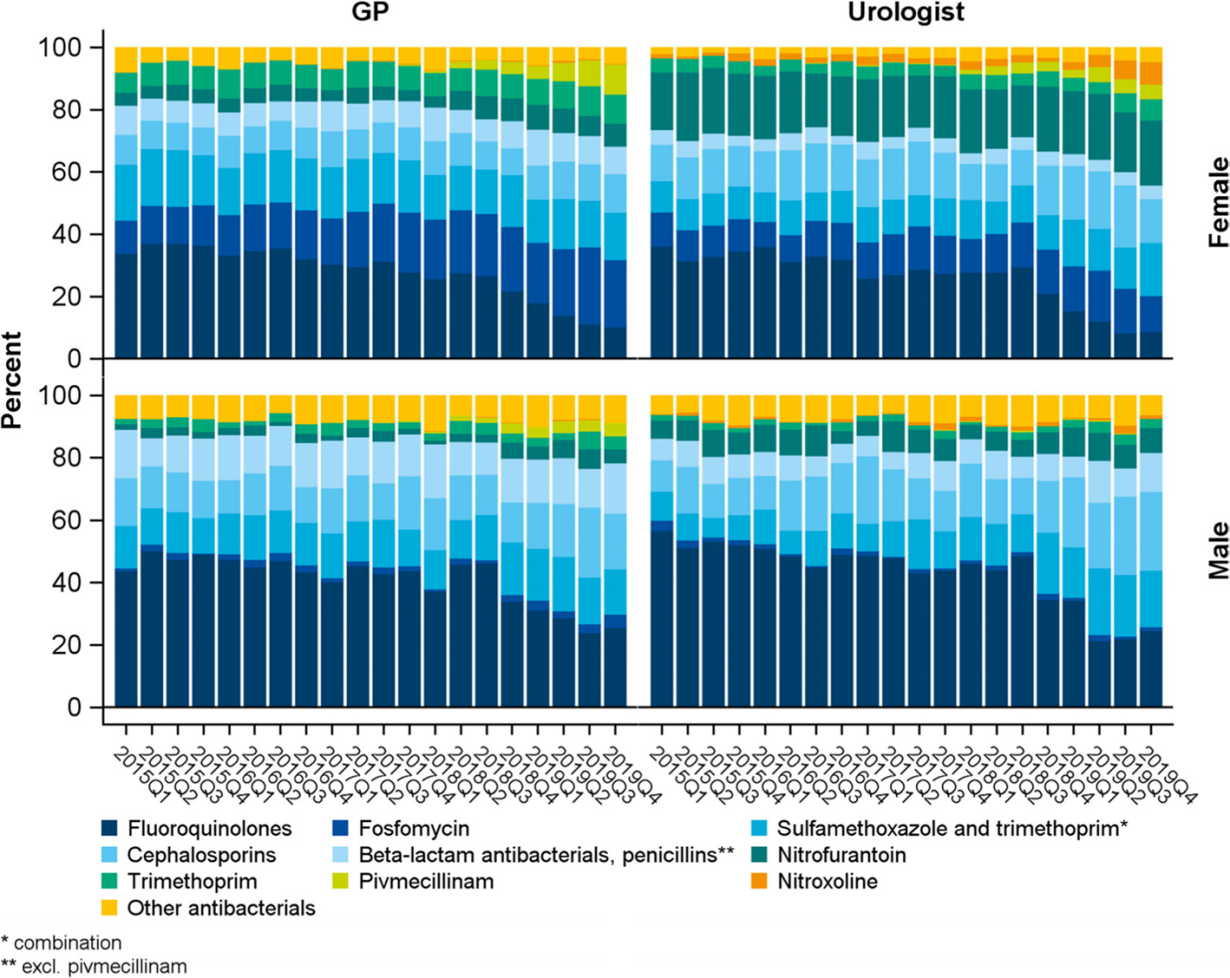
Trend: shares of antibacterials prescribed by GP and urologist in the quarter of a UTI diagnosis by sex. (*N* = 79,665 prescriptions)

GPs and urologists most often prescribed FQ (overall 29.9% and 33.0% of all antibacterial prescriptions, respectively). From the first quarter of 2015 to the last quarter of 2019, FQ prescriptions by GPs decreased from 35.4% to 12.7%, while fosfomycin increased (9.1% to 18.8%) as did pivmecillinam (up to 8.7%, Fig. 3). Shares of FQ prescriptions by urologists declined during the study period (from 43.3% to 14.7%), while use of co-trimoxazole increased (9.8% to 15.6%). The respective trends stratified by sex are displayed in Fig. 4.

Overall, nearly half of all antibacterial prescriptions issued by gynaecologists were fosfomycin (49.2%). Shares increased during the study period (from 44.6% to 52.3%, Fig. 3), while FQ prescriptions decreased (15.9% to 3.7%). Among paediatric prescriptions, cephalosporins dominated (overall 34.6%) although use decreased during the study period (43.6% to 26.1%). In contrast, penicillin prescriptions increased (18.4% to 26.4%) as did, to a lesser extent, co-trimoxazole and trimethoprim (mono preparation).

### Incident cases

In total, 38,099 cases had no UTI diagnoses in the 4 quarters preceding a respective quarter (Table 2). Incident cases were younger (median age 51 vs. 56 years) compared to all cases. They were also more often diagnosed by a GP (55.6% vs. 49.2%) and less often received diagnoses from a urologist (19.3% vs. 27.4%). Antibacterial treatment was more common with respect to any prescriptions (females: 79.7%, males: 69.5%) and prescriptions issued by a diagnosing specialty (females: 76.2%, males: 63.0%) compared to all cases. Additionally, in incident UTI cases, proportions of antibacterial treatment did not differ between age groups.

**Table 2:** Characteristics of incident UTI cases from 2016 to 2019 by sex and age group^a^ based on all incident UTI cases during the study period

	**Total**	**Sex**		**Age group**			
	**(*N* = 38,099)**	**Female (*N* = 29,483)**	**Male (*N* = 8,616)**	**0–5 years (*N* = 904)**	**6–13 years (*N* = 1,122)**	**14–17 years (*N* = 1,019)**	** > = 18 years** **(*N* = 35,054)**
**Age group in years**	**(*N* = 38,099)**	**(*N* = 29,483)**	**(*N* = 8,616)**	**(*N* = 904)**	**(*N* = 1,122)**	**(*N* = 1,019)**	**(*N* = 35,054)**
0–5	904 (2.4%)	715 (2.4%)	189 (2.2%)	904 (100%)			
6–13	1,122 (2.9%)	949 (3.2%)	173 (2.0%)		1,122 (100%)		
14–17	1,019 (2.7%)	925 (3.1%)	94 (1.1%)			1,019 (100%)	
18–24	3,888 (10.2%)	3,428 (11.6%)	460 (5.3%)				3,888 (11.1%)
25–34	5,506 (14.5%)	4,645 (15.8%)	861 (10.0%)				5,506 (15.7%)
35–44	4,121 (10.8%)	3,306 (11.2%)	815 (9.5%)				4,121 (11.8%)
45–54	4,288 (11.3%)	3,213 (10.9%)	1,075 (12.5%)				4,288 (12.2%)
55–64	4,258 (11.2%)	3,062 (10.4%)	1,196 (13.9%)				4,258 (12.1%)
65–74	4,424 (11.6%)	3,053 (10.4%)	1,371 (15.9%)				4,424 (12.6%)
75–84	5,858 (15.4%)	4,108 (13.9%)	1,750 (20.3%)				5,858 (16.7%)
85–94	2,481 (6.5%)	1,893 (6.4%)	588 (6.8%)				2,481 (7.1%)
95 +	230 (0.6%)	186 (0.6%)	44 (0.5%)				230 (0.7%)
**Diagnoses**	**(*N* = 38,099)**	**(*N* = 29,483)**	**(*N* = 8,616)**	**(*N* = 904)**	**(*N* = 1,122)**	**(*N* = 1,019)**	**(*N* = 35,054)**
N39.0	24,631 (64.6%)	18,737 (63.6%)	5,894 (68.4%)	760 (84.1%)	902 (80.4%)	693 (68.0%)	22,276 (63.5%)
N30.0	5,511 (14.5%)	4,158 (14.1%)	1,353 (15.7%)	42 (4.6%)	59 (5.3%)	101 (9.9%)	5,309 (15.1%)
N30.9	5,171 (13.6%)	4,351 (14.8%)	820 (9.5%)	65 (7.2%)	106 (9.4%)	148 (14.5%)	4,852 (13.8%)
N10	361 (0.9%)	292 (1.0%)	69 (0.8%)	10 (1.1%)	10 (0.9%)	4 (0.4%)	337 (1.0%)
Multiple diagnoses	2,425 (6.4%)	1,945 (6.6%)	480 (5.6%)	27 (3.0%)	45 (4.0%)	73 (7.2%)	2,280 (6.5%)
**Any antibacterial prescribed?**^b^	**(*N* = 38,099)**	**(*N* = 29,483)**	**(*N* = 8,616)**	**(*N* = 904)**	**(*N* = 1,122)**	**(*N* = 1,019)**	**(*N* = 35,054)**
Yes	29,476 (77.4%)	23,492 (79.7%)	5,984 (69.5%)	719 (79.5%)	881 (78.5%)	817 (80.2%)	27,059 (77.2%)
**Grouped diagnosing physician specialty**	**(*N* = 34,038)**	**(*N* = 26,314)**	**(*N* = 7,724)**	**(*N* = 804)**	**(*N* = 1,023)**	**(*N* = 934)**	**(*N* = 31,277)**
GP	18,925 (55.6%)	15,098 (57.4%)	3,827 (49.5%)	74 (9.2%)	174 (17.0%)	448 (48.0%)	18,229 (58.3%)
Urologist	6,573 (19.3%)	3,367 (12.8%)	3,206 (41.5%)	29 (3.6%)	77 (7.5%)	62 (6.6%)	6,405 (20.5%)
Gynaecologist	4,864 (14.3%)	4,852 (18.4%)	12 (0.2%)		16 (1.6%)	116 (12.4%)	4,732 (15.1%)
Paediatrician	1,739 (5.1%)	1,466 (5.6%)	273 (3.5%)	685 (85.2%)	727 (71.1%)	257 (27.5%)	70 (0.2%)
Other	453 (1.3%)	342 (1.3%)	111 (1.4%)	5 (0.6%)	3 (0.3%)	7 (0.7%)	438 (1.4%)
Multiple specialties	1,484 (4.4%)	1,189 (4.5%)	295 (3.8%)	11 (1.4%)	26 (2.5%)	44 (4.7%)	1,403 (4.5%)
**Any antibacterial prescribed by diagnosing specialty?**^c^	**(*N* = 34,038)**	**(*N* = 26,314)**	**(*N* = 7,724)**	**(*N* = 804)**	**(*N* = 1,023)**	**(*N* = 934)**	**(*N* = 31,277)**
Yes	24,862 (73.0%)	20,015 (76.1%)	4,847 (62.8%)	636 (79.1%)	797 (77.9%)	734 (78.6%)	22,695 (72.6%)

^a^no UTI diagnosed in the 4 quarters preceding the respective quarter

^b^in the quarter of a UTI diagnosis, by any physician

^c^in the quarter of a UTI diagnosis, by a physician specialty diagnosing a UTI in the respective quarter

*UTI* Urinary tract infection

Incident UTI cases received a total of 40,977 antibacterial prescriptions. Shares were comparable to those observed for all cases with 25.5% FQ, followed by fosfomycin (18.0%) and co-trimoxazole (15.6%, data not shown).

## Discussion

Based on the anonymized data from a regional SHI, prescribing patterns for 102,715 UTI cases over a five-year period in the federal state of Bremen were analysed. Prevalence of UTI remained stable with even a little decline (5.8 to 5.1%) in both sexes. The type of antibiotics prescribed was subject to relevant changes over time depending on the speciality.

The change in prevalence is in contrast to an increase reported from Norway between an earlier period [15]. Changes in antibiotic prescription with a general decline in antibiotic use for all indications was seen in Germany, mainly due to decreasing prescription rates in children. This decrease could be demonstrated for nearly all antibiotic classes with the exception of nitrofurantoin/ fosfomycin/nitroxoline for which the rates increased [16]. When focusing on UTI, FQ were still among the most common prescribed antibiotics but with a substantial decrease over recent years. Comparable data from other European countries report even lower rates of FQ prescriptions in urinary tract infections with 13.8% from Switzerland [17] or even 7% in Sweden [18]. The reasons for this trend towards a more guideline adherent prescription pattern in Germany are unclear. Guideline recommendations as well as Dear doctor letters regarding the restricted use of FQ might at least partly explain the changes. According to an earlier review, several approaches to improve antibiotic prescribing by healthcare providers in Primary Care were explored, but none of them could be identified as the most appropriate strategy [19]. A strategy that might explain the changes in our study is the development of local recommendations issued by an accepted body. Since 2011 General Practitioners in Bremen were provided with detailed evidence based recommendations [20]. They were developed by pharmacologists and supported by the local Association of Statutory Health Insurance Physicians and well as some SHI (including the one providing the data for this analysis). Their use is voluntary but supported by regular feedback and implementation of the recommendations in the physician’s software. From a previous study on uncomplicated urinary tract infections in north western Germany local resistance rates of bacteria are available, which support the antibiotic recommendations of the German guidelines [21].

Increasing evidence and subsequent guideline recommendations support the option of a non-antibiotic therapy in uncomplicated UTI in women [22] but not in men. In 2010 this option was mentioned in the German guideline and further endorsed in the 2017 update. Due to a lack of clinical data this approach is not recommended for men. Therefore, we would expect higher shares of antibiotic prescriptions in men when a UTI was diagnosed. However, the share of antibacterials prescribed was even lower in men (56.4%) than in women (70.8%). Even when including only incident cases (thus reducing possible documentation errors) this share increases only to 69.5% (women 79.7%). As a possible explanation, urologists more frequently use urine microscopy and will care for a higher number of patients with urinary catheters. This might result in higher detection rates of asymptomatic bacteriuria (due to the detection of leukocytes in otherwise asymptomatic patients). This condition should not be treated with antibiotics but might be coded as UTI. This is supported by the fact that 73.2% of all cases diagnosed by a GP received an antibiotic compared to 51.3% diagnosed by a urologist (data not shown). Another explanation might be that male patients were diagnosed and treated by a GP and additionally referred to a urologist. This is supported by the fact that 68.0% of male cases diagnosed by a GP received an antibiotic compared to 44.0% diagnosed by a urologist (data not shown). When patients have contacts with GPs and urologists within the same quarter an antibiotic prescription will be issued only by one specialty but the diagnosis will be coded by both, thus reducing the proportion of antibiotic prescriptions. And as another explanation, men with UTI seen in out of hours care will most probably receive an antibiotic prescription immediately. The diagnosis will be documented again, when seeing a GP or urologist afterwards but without another antibiotic prescription issued. We are not aware of any quality indicator or gold standard regarding an optimal prescriptions rate in relation to the number of diagnoses, therefore our data might foster further research and discussion on this topic.

Prescribing patterns between GPs, urologists and gynaecologists show remarkable differences regarding their antibiotic portfolio. Paediatricians differ from other disciplines as fosfomycin and FQ are hardly used. The first one being approved only from 12 years onwards, at this age the patients are mainly treated by GPs. FQ are only recommended as second line or in complicated infections, a recommendation that seems to be followed by paediatricians [23].

Pivmecillinam has been introduced in the German market in 2016 and is used mainly by GPs and gynaecologists with growing albeit still small prescription volumes. The reasons are probably the lack of experience with a new drug and at the beginning only sparse information regarding the resistance rates in Germany. In contrast, fosfomycin is increasingly used since its first recommendation in the 2010 guideline and the once only dose increases its acceptance with women. Fosfomycin is not licensed for men with UTI but when comparing its use between gynaecologists and GPs it becomes evident that gynaecologists prefer its use. Differences in the choice of antibiotics used have been described in the hospital setting [24] and between GPs [25] and GPs and paediatricians [26]. It is likely that resistance patterns of causative agents differ between the patients seen by specialists compared to those seen in primary care. FQ are still recommended in case of severe infections and therefore are likely to be more often used in selected patients with more complicated or advanced infections. However, as the German health care system allows direct access to specialists, a selection bias i.e. with more complicated or advanced infections seen by GPs is very unlikely. Probably there are more differences in prescribing culture between these specialties that have to be considered and probably can be of use to promote a higher adherence towards local or national guidelines.

### Strength and limitations

The main strength is the database covering all physician specialities involved in primary care and the large time period allowing an assessment of prescribing trends. Further, the AOK represents a high share of insured persons in the state of Bremen. Although insurance funds differ with respect e.g. to demographics, socio-economic status and morbidity [27], it seems unlikely that prescribing behaviour is influenced by affiliation with a certain SHI. Nevertheless, there are relevant regional differences regarding prescribing behaviour of antibiotics which limit a generalization of our results to different regions [16, 28]. Furthermore, contact patterns with GPs versus other specialists often differ between urban and rural areas- our results represent a consultation pattern in case of urinary tract infection that is likely to reflect the situation in an urban setting only. A further limitation is attributed to the administrative nature of the data: The quarterly reimbursement precludes determining whether multiple diagnoses in one or two subsequent quarters referred to one or more UTI cases. Accordingly, the prevalence of recurrent UTI remains imprecise. Another limitation is that no direct linkage is possible between prescriptions and diagnoses and therefore it cannot be ruled out that, in the quarter of a UTI diagnosis, antibacterials were prescribed for other infections. Due to the lack of clinical data (for example on renal function, allergies, further medication, comorbidities, results of urine cultures) a differentiation between complicated/uncomplicated infections is not possible. In complicated infections the use of second line antibiotics (e.g. FQ) is sometimes warranted. As we do not assume a relevant change in the share of complicated infections over time this limitation is unlikely to have an impact on our results.

## Conclusions

Despite a clear trend towards a more guideline adherent prescription pattern there is still room for improvement regarding the use of second line antibiotics especially fluoroquinolones. The choice of antibiotics prescribed differs between specialties with higher uptake of guideline recommended antibiotic by gynaecologists, mainly because of higher prescription shares of fosfomycin. An identification of individual prescribers was not possible due to the data structure. Such an analysis would be needed to identify those with higher proportions of inappropriate prescriptions. Based on our analysis we encourage every physician involved in treating patients with UTI to re-evaluate his or her portfolio of antibiotics used. A further reduction of second line antibiotics like cephalosporins seems feasible.

## Supplementary Information

**Additional file 1: Table S1*.*** Yearly UTI prevalences by sex and age group based on all persons insured for at last one day of the respective year*. ***Table S2*.*** Frequency of quarters with at least one UTI diagnosis on a person level based on all persons with at least one UTI during the study period*.*

## Data Availability

The datasets generated and/or analysed during the current study are not publicly available but are available from the corresponding author on reasonable request and with permission of the AOK Bremen/ Bremerhaven.

## Abbreviations

ATC: Anatomical therapeutic chemical
FC: Fluoroquinolone
GP: General practitioner
SHI: Statutory health insurance
N 39.0: Urinary tract infection, site not specified
N30.0: Acute cystitis
N30.9: Cystitis, unspecified
N10: Acute tubulo-interstitial nephritis
