# Supplementary material for: Patterns and trends of antibacterial treatment in patients with urinary tract infections, 2015–2019: an analysis of health insurance data
Source: BMC Prim Care. 2022 Aug 11;23:204. doi: 10.1186/s12875-022-01816-6 (PMC9367112; doi:10.1186/s12875-022-01816-6)
Supplement: Supplementary file 1 — Additional file 1: Table S1. Yearly UTI prevalences by sex and age group based on all persons insured for at last one day of the respective year. Table S2. Frequency of quarters with at least one UTI diagnosis on a person level based on all persons with at least one UTI during the study period. [file 12875_2022_1816_MOESM1_ESM.docx]

**Supplement**

**Supplemental Table 1: Yearly UTI prevalences by sex and age group based on all persons insured for at last one day of the respective year**

|  | **2015** | **2016** | **2017** | **2018** | **2019** |
| --- | --- | --- | --- | --- | --- |
| **Total** | 5.8% | 5.7% | 5.6% | 5.3% | 5.1% |
| **By sex** |  |  |  |  |  |
| Female | 9.2% | 9.0% | 9.0% | 8.6% | 8.2% |
| Male | 2.5% | 2.4% | 2.4% | 2.2% | 2.1% |
| **By age group in years** |  |  |  |  |  |
| 0-5 | 2.0% | 1.7% | 1.8% | 1.6% | 1.4% |
| 6-13 | 1.9% | 1.7% | 1.7% | 1.5% | 1.5% |
| 14-17 | 3.5% | 3.6% | 3.1% | 3.0% | 2.6% |
| 18-24 | 6.6% | 6.4% | 6.3% | 5.8% | 5.6% |
| 25-34 | 5.6% | 5.6% | 5.7% | 5.2% | 5.1% |
| 35-44 | 5.2% | 5.0% | 5.2% | 5.1% | 4.7% |
| 45-54 | 4.5% | 4.6% | 4.7% | 4.6% | 4.6% |
| 55-64 | 5.5% | 5.4% | 5.4% | 5.2% | 5.0% |
| 65-74 | 7.6% | 7.6% | 7.6% | 7.5% | 7.2% |
| 75-84 | 11.2% | 11.0% | 11.0% | 10.5% | 10.0% |
| 85-94 | 12.7% | 12.5% | 12.6% | 11.9% | 12.1% |
| 95+ | 12.0% | 12.3% | 14.0% | 11.5% | 11.4% |

UTI: urinary tract infection

**Supplemental Table 2: Frequency of quarters with at least one UTI diagnosis on a person level based on all persons with at least one UTI during the study period**

|  | **Total  (N=47,396)** | **Female  (N=36,330)** | **Male  (N=11,066)** |
| --- | --- | --- | --- |
| Number of quarters with at least one UTI diagnosis |  |  |  |
| 1 | 27,842 (58.7%) | 20,399 (56.2%) | 7,443 (67.3%) |
| 2 | 9,083 (19.2%) | 7,291 (20.1%) | 1,792 (16.2%) |
| 3-4 | 6,154 (13.0%) | 5,159 (14.2%) | 995 (9.0%) |
| 5-9 | 3,168 (6.7%) | 2,618 (7.2%) | 550 (5.0%) |
| 10+ | 1,149 (2.4%) | 863 (2.4%) | 286 (2.6%) |
| UTI diagnoses in two consecutive quarters | 10,431 (22.0%) | 8,245 (22.5%) | 2,186 (19.8%) |
| UTI diagnoses in three of four consecutive quarters | 5,385 (11.4%) | 4,245 (11.7%) | 1,140 (10.3%) |

UTI: urinary tract infection
